# Intestinal fatty acid binding protein as a marker for intra-abdominal pressure-related complications in patients admitted to the intensive care unit; study protocol for a prospective cohort study (I-Fabulous study)

**DOI:** 10.1186/s13049-015-0088-0

**Published:** 2015-01-16

**Authors:** Steven G Strang, Oscar J F Van Waes, Ben Van der Hoven, Samir Ali, Michael H J Verhofstad, Peter Pickkers, Esther M M Van Lieshout

**Affiliations:** Trauma Research Unit Department of Surgery, Erasmus MC, University Medical Center Rotterdam, P.O. Box 2040, 3000 CA Rotterdam, The Netherlands; Department of Intensive Care Medicine, Erasmus MC, University Medical Center Rotterdam, P.O. Box 2040, 3000 CA Rotterdam, The Netherlands; Department of Intensive Care Medicine, Radboud University Medical Center, Nijmegen, The Netherlands

**Keywords:** Abdominal Compartment Syndrome, Biomarker, Critical care medicine, Intensive Care Unit, Intestinal integrity, Intra-abdominal hypertension, Intra-abdominal pressure, Risk factors

## Abstract

**Background:**

Intra-abdominal hypertension (IAH) and abdominal compartment syndrome (ACS) have detrimental effects on all organ systems and are associated with increased morbidity and mortality in critically ill patients admitted to an intensive care unit. Intra-bladder measurement of the intra-abdominal pressure (IAP) is currently the gold standard. However, IAH is not always indicative of intestinal ischemia, which is an early and rapidly developing complication. Sensitive biomarkers for intestinal ischemia are needed to be able to intervene before damage becomes irreversible. Gut wall integrity loss, including epithelial cell disruption and tight junctions breakdown, is an early event in intestinal damage. Intestinal Fatty Acid Binding Protein (I-FABP) is excreted in urine and blood specifically from damaged intestinal epithelial cells. Claudin-3 is a specific protein which is excreted in urine following disruption of intercellular tight junctions. This study aims to investigate if I-FABP and Claudin-3 can be used as a diagnostic tool for identifying patients at risk for IAP-related complications.

**Methods/Design:**

In a multicenter, prospective cohort study 200 adult patients admitted to the intensive care unit with at least two risk factors for IAH as defined by the World Society of the Abdominal Compartment Syndrome (WSACS) will be included. Patients in whom an intra-bladder IAP measurement is contra-indicated or impossible and patients with inflammatory bowel diseases that may affect I-FABP levels will be excluded. The IAP will be measured using an intra-bladder technique. During the subsequent 72 hours, the IAP measurement will be repeated every six hours. At these time points, a urine and serum sample will be collected for measurement of I-FABP and Claudin-3 levels. Clinical outcome of patients during their stay at the intensive care unit will be monitored using the Sequential Organ Failure Assessment (SOFA) score.

**Discussion:**

Successful completion of this trial will provide evidence on the eventual role of the biomarkers I-FABP and Claudin-3 in predicting the risk of IAP-associated adverse outcome. This may aid early (surgical) intervention.

**Trial registration:**

The trial is registered at the Netherlands Trial Register (NTR4638).

**Electronic supplementary material:**

The online version of this article (doi:10.1186/s13049-015-0088-0) contains supplementary material, which is available to authorized users.

## Background

Patients undergoing major surgery or sustaining severe trauma are at risk of developing morbidity and mortality from postoperative or posttraumatic systemic inflammatory response syndrome, sepsis and multiple organ dysfunction. The development of such potentially lethal complications in otherwise healthy patients is poorly understood. Data from a prospective multicenter epidemiological study showed that intra-abdominal hypertension (IAH) is associated with increased morbidity and mortality rates in critically ill patients admitted to an intensive care unit (ICU) [1,2]. Therefore, early identification of patients at risk for IAH-related morbidity and mortality can be potentially lifesaving.

### Intra-abdominal pressure and intra-abdominal hypertension

Compliance of the abdominal wall together with the abdominal content determines the intra-abdominal pressure (IAP) level. Under physiologic situations, the IAP level is below 12 mmHg. Increased pressure in the abdomen is known as intra-abdominal hypertension (IAH). This is defined as a sustained IAP ≥12 mmHg [3]. IAH results in reduced blood flow to most organs, with consequent dysfunction of the cardiovascular, respiratory, renal, gastrointestinal, and central nervous systems. An IAP >20 mmHg in combination with new organ dysfunction is indicative of an abdominal compartment syndrome (ACS). The ultimate treatment of ACS is a decompressive laparotomy. Risk factors for ACS include leakage of an aneurysm of the abdominal aorta, closing the abdomen under pressure after abdominal surgery, damage control laparotomy, hyperhydration during hypovolemic shock, pancreatitis, and pulmonary contusion. These risk factors may apply to a majority of patients in the ICU. The mortality risk in patients with ACS may be as high as 80% [1].

The current gold standard measurement tool as put forward by the World Society of the Abdominal Compartment Syndrome (WSACS; http://www.wsacs.org/education/algorithms.html) is an intra-bladder pressure measurement. This is a simple, minimally invasive method, and the results are immediately available [3]. From a clinical perspective, however, the IAP level does not always represent the presence of intestinal ischemia and as such is not a perfect indicator for clinical outcome nor surgical therapy. Sensitive biomarkers indicative of early (*i.e.*, reversible) organ dysfunction are therefore needed as additional diagnostic tool. The combination of increased IAP and a biomarker level that represents organ damage would support the need for and timing of decompressive measures to relieve the abdominal pressure.

### Biomarkers released upon loss of small intestinal integrity

Evidence is accumulating that the intestines play a central role in the origin of postoperative and posttraumatic sequelae [4-6]. Enterocyte damage and tight junction loss can be triggered by IAH, and both result in loss of intestinal integrity. As a consequence, toxins, bacteria, and undigested food particles may pass the enterocyte layer, enter the underlying vasculature, and trigger systemic inflammatory reactions that may progress to multiple organ dysfunction syndrome and even death. Peptides released upon enterocyte damage (*e.g.*, Intestinal Fatty Acid Binding protein; I-FABP) or tight junction loss (*e.g.*, Claudin-3) are potentially ideal biomarkers that will help identify patients with early IAH-induced intestinal damage.

I-FABP is a small (14–15 kDa) protein that is exclusively present in mature enterocytes of the small and large intestine. It is released into the circulation upon enterocyte membrane integrity loss and rapidly excreted into the urine (half-life 11 minutes). Elevated I-FABP levels have been found in plasma, serum, and urine in patients with intestinal ischemia, celiac disease, systemic inflammatory response syndrome and necrotizing enterocolitis [7-15]. I-FABP levels can be measured sensitively in plasma, serum, and urine using an enzyme-linked immunosorbent assay (ELISA) [7,8].

Claudins are small (22 kDa) tight junction proteins [4,16-19]. Especially Claudin-3 is expressed in high quantities solely in the intestine [18]. The amount of Claudin-3 in the intestine decreases after tight junction integrity loss [17]. Breakdown of tight junctions by loss of Claudins (as measured on Western blots in urine) is an early event in intestinal damage, resulting in intestinal barrier loss [20].

The main aim of the current study is to investigate if urinary levels of I-FABP can be used as a diagnostic tool for identifying patients at risk for IAP-related complications. Secondary aims are to determine the same for serum levels of I-FABP and for urinary and serum levels of Claudin-3.

## Methods/Design

### Study design and setting

Multicenter prospective observational study of 200 patients admitted to the ICU of Erasmus MC, University Medical Center Rotterdam (Rotterdam, The Netherlands) or Radboud University Medical Center Nijmegen (Nijmegen, The Netherlands), who are at risk for developing IAH or ACS according to the definitions of the World Society of the Abdominal Compartment Syndrome [3]. Since it was suggested that patients who have undergone liver transplantation should also be screened for IAH [21,22], liver transplantation has been added to the list of risk factors. The study is registered at the Netherlands Trial Register (NTR4638).

### Study population and eligibility criteria

All adult patients admitted to the ICU who have at least two risk factors for developing IAH or ACS according to the WSACS will be eligible for inclusion [3].

Patients meeting the following inclusion criteria will be eligible for enrolment:

1. Patients with at least two risk factors putting them at risk for IAH or ACS as agreed by the WSACS. Risk factors may be present already at admission, but also patients developing risk factors during ICU stay will be eligible from that moment onwards;
2. Age 18 or older, with no upper age limit;
3. Signed informed consent by patient or proxy.

If any of the following criteria applies, patients will be excluded:

1. Patients in whom intra-bladder pressure measurement is contra-indicated. This includes patients with bladder trauma or hematuria;
2. Patients in whom intra-bladder pressure measurements are not reliable due to intraperitoneal adhesions, bladder oppressive pelvic hematoma, abdominal packs in situ, (previous) bladder tumor or previous bladder removal;
3. Patients with any inflammatory bowel disease that may affect I-FABP levels.

Exclusion of a patient because of enrolment in another ongoing drug or surgical intervention trial will be left to the discretion of the attending surgeon on a case-by-case basis.

### Treatment

Patients with increased pressure determined by the intra-bladder pressure measurements will be treated in compliance with the algorithms for patient management as developed by the WSACS (www.wsacs.org/algorithms.php) [3]. The IAH Assessment Algorithm describes all risk factors that every patient should be screened for upon intensive care unit admission and upon clinical deterioration leading to new organ dysfunction. The intra-bladder pressure will be measured in patients with two or more risk factors for IAH or ACS or in patients who have undergone a liver transplantation. Patients who do not have two or more risk factors at baseline but develop new risk factors during their ICU admission will be followed from that moment onwards. The reason for this is that the occurrence of IAH during intensive care stay is known to be an independent predictor for mortality, whereas presence of IAH at intensive care admission is not [23]. Patients should be enrolled as soon as possible, but at least within 48 hours after meeting the eligibility criteria. The IAH/ACS Management Algorithm provides a decision tree for the follow-up of patients related to the IAP level. The IAH/ACS Medical Management Algorithm provides a stepwise approach of actions to be taken for achieving IAP pressure relief. In short, ICU management will be provided by the attending physicians and may consist of, among others, balanced intravenous fluid administration, correction of hypovolemia, electrolyte disturbances and/or anemia and analgesics.

### Outcome measures

I-FABP levels will serve as primary outcome measure. Urinary and serum concentrations of I-FABP will be analyzed in duplicate using a highly specific, commercially available enzyme-linked immunosorbent assay (ELISA) that selectively detects human I-FABP (HyCult Biotechnology, Uden, The Netherlands). The ELISA will be performed following the supplier’s protocol. I-FABP levels in urine will be adjusted to urinary creatinine levels.

Claudin-3 levels will serve as secondary outcome measure. Urinary levels of Claudin-3 will be analyzed in duplicate either by ELISA (if available at time of analysis) or by Western blotting. Claudin-3 levels in urine will be adjusted to urinary creatinine levels. If analyzed from Western blots, equal amounts of each sample (adjusted to urinary creatinine levels) will be separated by sodium dodecyl sulphate polyacrylamide gel electrophoresis (SDS-PAGE), transferred to a polyvinylidene fluoride (PVDF) membrane and probed using primary antibody to Claudin-3 (Rabbit anti-claudin-3 (34–1700), Zymed Laboratories, San Francisco, CA). After incubation with goat anti rabbit HRP-conjugated secondary antibody (Jackson, West Grove, PA), a signal will be detected by supersignal west pico chemiluminescence substrate (Pierce, Etten-Leur, the Netherlands). Band intensities for each sample will be semi-quantitatively analyzed using Quantity One (Biorad, Hercules, CA). This is a straightforward approach for semi-quantitative assessment of protein levels. Urinary creatinine levels in the collected samples will be determined at the Clinical Chemistry department.

The occurrence of (increasing) organ dysfunction will be based upon the SOFA score.

### Baseline, disease and treatment-related data

In addition to the outcome variables mentioned above, the following data will be collected:

***Intrinsic variables (baseline data)***:

- Age
- Gender
- Weight
- Height
- Comorbidity

***Disease-related variables at admission***:

- Reason of admission to the ICU (*i.e.*, type of disease or injury and type of intervention)
- Serum lactate concentration at baseline (sodium fluoride tube)
- IAP level at baseline
- Urinary creatinine level at baseline (sediment tube)
- Acute Physiology and Chronic Health Evaluation II (APACHE II) score at baseline; this is a validated and commonly applied severity of disease classification system [24]. An integer score ranging from 0 to 71 is computed based upon 12 routine physiological measurements with a higher scores implying a more severe disease and a higher risk of death.
- Acute Physiology and Chronic Health Evaluation IV (APACHE IV) score at baseline; this standardized scoring metrics was developed in 2005 as an improved version of the APACHE II. The previously used set of equations were re-evaluated and improved where needed. The most important change involved the new categorization of disease groups [25].
- Simplified Acute Physiology Score II (SAPS II) at baseline; this score was designed to measure the severity of disease for patients admitted to Intensive care units aged 15 or more [26]. The SAPS II score is calculated from 12 routine physiological measurements during the first 24 hours of ICU admission, information about previous health status and some information obtained at admission. The computed score has a range from 0 to 163 points and a predicted mortality between 0% and 100%. Higher scores imply a more severe disease and a higher risk of death.
- Sequential Organ Failure Assessment (SOFA) score at baseline; this score is a validated and commonly used score to track a patient’s status during the stay in an ICU. The SOFA score is a scoring system to determine the extent of a person’s organ function or rate of failure [27-31]. The score is based upon six different scores, one each for the respiratory system (*i.e.*, PaO2/FiO2 ratio), the cardiovascular system (*i.e.*, mean arterial pressure and vasopressor requirement), the hepatic system (*i.e.*, bilirubin level), the coagulation system (*i.e.*, platelet count), the renal system (*i.e.*, creatinine level or urine output), and the neurological system (*i.e.*, Glasgow Coma Score). For each of these systems, a maximum of four points can be attributed. SOFA scores are determined on a daily basis during ICU admission.

***Treatment-related variables***:

- Medication use
- Interventions performed (*i.e.*, type and number of interventions)
- Sequential Organ Failure Assessment (SOFA) score on day 1 to 4 (or until ICU discharge or death).
- Length of stay in the ICU
- Mortality during ICU stay and during hospital stay

### Sample size calculation

In order to reliably calculate correlation coefficients for the association between IAP, I-FABP and Claudin-3 levels, at least 75 patients with IAH are needed. Malbrain *et al.* showed in a multicenter study that in a general ICU population, the prevalence of IAH (*i.e.*, IAP > 12 mmHg) and ACS (*i.e.*, IAP > 20 mmHg with concomitant new organ dysfunction) was 32%, and 4%, respectively [23]. Since we will only follow-up on patients with a minimum of two IAH/ACS risk factors, the percentage of enrolled patients that will develop IAH or ACS will be higher, and is likely to exceed 40% (unpublished data). Therefore, enrolling a total population of 200 patients will be sufficient to ascertain availability of 80 patients with IAH.

This will also be sufficient to compare I-FABP levels in patients with physiologic IAP levels with patients that developed IAH with adequate statistical power. Based upon data provided by Kanda *et al*. we expect that mean I-FABP levels will be 20.0 ± 10.0 ng/mL (range 0–100 ng/mL) in patients without IAH [32]. In patients with IAH, the I-FABP levels will be higher (≥20 ng/mL). An overall population of 200 patients (consisting of 120 controls and 80 patients with IAH) will be sufficient to detect a 0.5 SD increase to 30.0 ± 12.5 ng/mL in I-FABP level in patients with IAH (two-sided test with an α level of 0.05) with >90% statistical power.

### Recruitment and consent

Upon identification of two IAH risk factors, treatment should be initiated in compliance with international guidelines of the WSACS. This implies that the baseline IAP measurement and the first urine sample may have been collected prior to obtaining informed consent from the patient or his/her legal representative. Eligible persons admitted to the ICU who are at risk for developing IAH will be informed about the trial and asked for consent at the ICU. If patients are unconscious or otherwise not able to sign informed consent, their legal representative will be informed about the trial and asked to sign informed consent on behalf of the patient. Upon recovery the patient will be asked to sign final consent. The patient and their legal representative will receive information and a consent form from the attending physician, the clinical investigator or a research assistant. If a patient or his/her legal representative decides not to sign informed consent, data and samples collected for that patient will be disposed of, and patients will be excluded from analysis.

### Study procedures

In patient with ≥2 risk factors for IAH or ACS, the intra-abdominal pressure will be measured using an intra-bladder technique. The modified Kron technique described by Cheatham and Safcsak [33] will be applied for measuring IAP. In the current study, 20 mL of saline will be used in order to comply with the current recommendations of the WSACS. For the IAP measurement, a Foley catheter will be disconnected and a 3-way-valve will be inserted to create a continuous connection to a pressure transducer (DTXPlus™ PRESS PA, reference No 686496; Argon Critical Care Systems, Singapore). For every single measurement the valve connecting the urinary drainage bag is closed and 20 mL of saline is instilled. The midaxillary line will be used as reference.

During the first 72 hours after enrolment, IAP measurement will be repeated every six hours (Table 1). At those time points (including baseline), the following samples will be collected:

**Table 1:** **Schedule of enrolment, interventions, and assessments**

	**Study period**		
	Enrolment*	Measurement **	**Close-out**
**TIMEPOINT**	***<T***_***0***_	***T***_***0***_ ***to T***_***72***_	***ICU discharge***
**ENROLMENT:**			
**Eligibility screen**	X		
**Informed Consent**	X		
**ASSESSMENTS:**			
**Baseline variables**	X		
**Disease-related variables**	X		
**IAP measurement**		X	
**Blood sample**		X	
**Urine sample**		X	
**I-FABP measurement**		X	
**Claudin-3 measurement**		X	
**Creatinine measurement**		X	
**Treatment-related variables**		X	
**SOFA score**		X	
**Adverse events**		X	X
**Length of ICU stay**		X	X
**Mortality during ICU stay**		X	X
**Secondary interventions**		X	X

*Patients are considered eligible as soon as two IAH risk factors are present. This can either be at ICU admission or later on during ICU stay.

**Patients are followed until 72 hours or until discharge from the ICU, whichever comes first. Measurements are performed every six hours.

1. Urine samples for measurement of I-FABP, Claudin-3 and creatinine levels: A single fresh specimen of urine will be collected from the urinary bladder catheter that is already in situ. Samples will be kept on ice and then frozen at −80°C in aliquots within two hours of collection.
2. Blood samples for measurement of serum I-FABP levels (NB: this only applies to patients enrolled at Erasmus MC, Rotterdam, the Netherlands): A single blood sample (10 mL) will be drawn from the arterial line that is already *in situ*. Blood will be collected in pre-chilled vacutainer containing EDTA as anticoagulent (BD Vacutainer, Becton Dickinson Diagnostics, Aalst, Belgium) and kept on ice. Blood will be centrifuged at 4°C, 4000× g for 15 minutes. Serum will be stored in aliquots at −80°C within 2 hours until analysis.

The reason for following up on patients during the first 72 hours after enrollment is based upon our observation (unpublished data) that over 95% of patients that deteriorate clinically due to increased IAP will do so during the 12–24 hours. In order not to miss any patients that may deteriorate somewhat later (*e.g.*, due to leak at the surgical site), we have set the time frame at 72 hours.

In order to assess if levels of I-FABP or Claudine-3 can be used as a prognostic marker for intestinal ischemia-related morbidity it is necessary to also collect clinical data of patients during their entire stay at the ICU. These data will be extracted from the ICU patient data management system (PDMS), where they are stored as part of clinical routine. This will be done using the APACHE II, APACHE IV, SAPS II and SOFA score, which will be collected routinely for any patient admitted to the ICU. Follow-up in the ICU will include a daily physical examination, vital signs monitoring, routine blood tests, and chest radiographs or other ancillary tests as required. The attending physicians will record complications and events as well as any (secondary) intervention performed such as decompression laparotomy.

### Data collection

Most variables will be collected as part of standard of care and are routinely recorded in electronic patients records. The intra-abdominal pressure will be measured by the ward nurse and recorded in the PDMS system and on case record forms (CRF). This CRF contains no patient identifiers. Urine and serum samples will be collected, processed and frozen at −80°C by the researchers or a research nurse. I-FABP and Claudin-3 levels will be measured by the researcher (SGS) and results will be recorded in the CRF. CRFs are stored and secured at the hospital where the patient is included.

### Data management

Research data will be stored in a database (SPSS), and will be handled confidentially and anonymously. Research data that can be traced to individual patients can only be viewed by authorized personnel. These persons are the members of the research team, members of the health care inspection, and members of the Medical Ethics Committee Erasmus MC. The handling of personal data will be in compliance with the Dutch Data Protection Act and the privacy regulation of the Erasmus MC.

Research data will be stored under a code number. Only the code number will be used for study documentation, progress reports and publications. The principal investigator and research assistant are the only persons with the information linking individual persons to study code numbers. Patient data and materials will be stored for a maximum of 15 years after the end of the study. Patients need to consent with this, and if not, their materials will be disposed of upon termination of the study.

### Statistical analysis

Data will be analyzed at the end of the study using the Statistical Package for the Social Sciences (SPSS) version 21 or higher (SPSS, Chicago, Ill., USA) and will be reported following the STrengthening the Reporting of OBservational studies in Epidemiology (STROBE) guidelines. No interim analysis will be done. Normality of continuous data will be assessed by the Shapiro Wilk tests and by inspecting the frequency distributions (histograms). Homogeneity of variances will be tested using the Levene’s test.

Descriptive analysis will be performed in order to report baseline characteristics (intrinsic variables and disease-related variables) and outcome measures of the entire cohort as well as for the group of patients that develop IAH (IAH group; IAP of 12 mmHg or higher on two separate recordings) versus those that do not (control group). For continuous data mean and SD (parametric data) or medians and percentiles (non-parametric data) will be calculated and reported. For categorical data, numbers and frequencies will be calculated and reported for the entire cohort and for the IAH and the control group separately.

Univariate analysis will be performed in order to test the difference in the primary and secondary outcome measures between the IAH group and the control group. Continuous data such as the I-FABP level (primary outcome) of the Claudin-3 level (secondary outcome) will be tested using a Student’s *T*-test (parametric data) or a Mann Whitney *U*-test (non-parametric data). Chi-squared analysis will be used for statistical testing of categorical data such as the number of secondary interventions. A p-value <0.05 will be taken as threshold of statistical significance. Depending upon the number of patients within the cohort of the study that develop ACS and the mortality rate, similar tests will be used to compare data for those who developed ACS (or those who died) versus those who did not. The patient cohort to be included will be heterogeneous by nature, as IAH and ACS risk factors will differ between patients. If it is possible to identify subgroups of patients (*e.g.*, liver transplant patients) subgroup analyses may be performed. P-values <0.05 will be taken as threshold of statistical significance.

The prognostic role of the I-FABP and Claudin-3 on development of IAH, ACS, (increasing) organ dysfunction (based upon SOFA increase) or mortality will be assessed. This analysis will be performed with Stata software, version 10.0 or higher (StatCorp LP, College Station, TX, USA), using the generalized linear latent and mixed model (GLLAMM) framework. Herein, the binary outcome IAH (or new organ dysfuncion or ACS or mortality) will be used as dependent variable, and the level of I-FABP or Claudin-3 will be included in the model as time-dependent variable. Patient ID will be included as clustering variable as up to 13 measurements per patient will be available. Additional covariates such as age, gender, number of IAH risk factors, and BM will be entered into the model in order to evaluate their effect on the relation between biomarker level and outcome. Random intercept and slope will be considered. Results will be expressed as Odds Ratios with their corresponding 95% confidence interval and p-values.

### Data monitoring

No data monitoring committee has been established as this study is not an interventional study and all data are either recorded in patient files (*i.e.*, all clinical data including baseline, disease-related and treatment-related variables) as part of standard of care, or are generated electronically (*i.e.,* ELISA results and laboratory tests). A random sample of at least 10% of all data will be double checked by a member of the research team in order to check the quality of the data entry into the database. The only exception to this will be the primary outcome, for which 100% of data will be checked.

### Ethical considerations

The study will be conducted according to the principles of the Declaration of Helsinki (64^th^ World Medical Association General Assembly, Fortaleza, October 2013). This study has been given a waiver by the medical research ethics committee (MREC) Erasmus MC, University Medical Center Rotterdam (Rotterdam, The Netherlands; reference number MEC-2011-016) and by the local hospital board in the participating center (Radboud University Medical Center, Nijmegen, The Netherlands). Following review of the protocol (version 1.0, dd December 13, 2010), the MREC concluded that this study is not subject to the Medical Research Involving Human Subjects Act (WMO, in Dutch “Wet Medisch-wetenschappelijk Onderzoek met mensen”). They concluded that the study is a medical/scientific research, but no patients are subjected to procedures or are required to follow rules of behavior. Consequently, the statutory obligation to provide insurance for subjects participating in medical research (article 7, subsection 6 of the WMO and Medical Research (Human Subjects) Compulsory Insurance Decree of 23 June 2003) was also waived. The reason for this dispensation is that participation in this study is without risks. Any important protocol amendments will be submitted to the MREC Erasmus MC before implementation.

### Dissemination policy

Research data can be presented or publicized in agreement with the clinical investigator and project leaders only. No research data that can be traced to individual persons will be presented or published.

## Discussion

Every year, approximately 80,000 patients are admitted to an ICU in the Netherlands [34]. Specific groups of patients such as those who underwent major surgery (*e.g.*, vascular or intestinal surgery) or who sustained severe trauma are at risk of developing morbidity and mortality from postoperative or posttraumatic systemic inflammatory response syndrome, sepsis and multiple organ dysfunction. The development of such potentially lethal complications in relatively healthy surgical or trauma patients is poorly understood. Evidence is accumulating that intra-abdominal hypertension plays a central role in the origin of such postoperative and posttraumatic sequelae. An increased intra-abdominal pressure level is currently the best indicator of intra-abdominal complications. However, the IAP level does not always represent the presence or absence of organ dysfunction, substantial intra-abdominal damage may be present already prior to the development of IAH and ACS. There is a distinctive need for a non-invasive early-onset diagnostic test and biomarkers. The main aim of the current study is to test if urinary I-FABP or Claudine-3 levels can be used as diagnostic tool for identifying patients at risk for IAP-related complications. This marker can then be used in order to take the necessary clinical measures (*e.g.*, decompressive laparotomy) before the IAP has reached the level of irreversible damage to vital organs. This may prevent serious morbidity or even death in a large group patients admitted to the ICU.

### Trial status

The study began enrolling patients on April 18, 2011.

## Abbreviations

ACS: Abdominal compartment syndrome
APACHE II: Acute physiology and chronic health evaluation II
APACHE IV: Acute physiology and chronic health evaluation IV
ELISA: Enzyme-linked immunoSorbent assay
HRP: Horse-radish peroxidase
IAH: Intra-abdominal hypertension
IAP: Intra-abdominal pressure
ICU: Intensive care unit
I-FABP: Intestinal fatty acid binding protein (I-FABP)
METC: Medisch ethische toetsings commissie
MREC: Medical research ethics committee
NTR: Netherlands trial register
PVDF: Polyvinylidene fluoride
SAPS II: Simplified acute physiology score II
SDS-PAGE: Sodium dodecyl sulphate polyacrylamide gel electrophoresis
SOFA: Sequential organ failure assessment
SPSS: Statistical package for the social sciences
STROBE: STrengthening the reporting of OBservational studies in epidemiology
WMO: Wet medisch-wetenschappelijk Onderzoek met mensen
WSACS: World society of the abdominal compartment syndrome (WSACS)
